# CPP-ZFN: A potential DNA-targeting anti-malarial drug

**DOI:** 10.1186/1475-2875-9-258

**Published:** 2010-09-16

**Authors:** Vikrant Nain, Shakti Sahi, Anju Verma

**Affiliations:** 1 School of Biotechnology, Gautam Buddha University, Greater Noida-201308, India; 2 School of Biological Sciences, University of Missouri, Kansas City, MO- 64110, USA

## Abstract

**Background:**

Multidrug-resistant *Plasmodium *is of major concern today. Effective vaccines or successful applications of RNAi-based strategies for the treatment of malaria are currently unavailable. An unexplored area in the field of malaria research is the development of DNA-targeting drugs that can specifically interact with parasitic DNA and introduce deleterious changes, leading to loss of vital genome function and parasite death.

**Presentation of the hypothesis:**

Advances in the development of zinc finger nuclease (ZFN) with engineered DNA recognition domains allow us to design and develop nuclease of high target sequence specificity with a mega recognition site that typically occurs only once in the genome. Moreover, cell-penetrating peptides (CPP) can cross the cell plasma membrane and deliver conjugated protein, nucleic acid, or any other cargo to the cytoplasm, nucleus, or mitochondria. This article proposes that a drug from the combination of the CPP and ZFN systems can effectively enter the intracellular parasite, introduce deleterious changes in its genome, and eliminate the parasite from the infected cells.

**Testing the hypothesis:**

Availability of a DNA-binding motif for more than 45 triplets and its modular nature, with freedom to change number of fingers in a ZFN, makes development of customized ZFN against diverse target DNA sequence of any gene feasible. Since the *Plasmodium *genome is highly AT rich, there is considerable sequence site diversity even for the structurally and functionally conserved enzymes between *Plasmodium *and humans. CPP can be used to deliver ZFN to the intracellular nucleus of the parasite. Signal-peptide-based heterologous protein translocation to *Plasmodium*-infected RBCs (iRBCs) and different *Plasmodium *organelles have been achieved. With successful fusion of CPP with mitochondrial- and nuclear-targeting peptides, fusion of CPP with 1 more *Plasmodium *cell membrane translocation peptide seems achievable.

**Implications of the hypothesis:**

Targeting of the *Plasmodium *genome using ZFN has great potential for the development of anti-malarial drugs. It allows the development of a single drug against all malarial infections, including multidrug-resistant strains. Availability of multiple ZFN target sites in a single gene will provide alternative drug target sites to combat the development of resistance in the future.

## Background

Malaria is the most devastating human parasitic infection. It threatens half of the world's population, killing more than 1 million people each year [1,2]. Malaria species vary widely in epidemiology and clinical manifestation [3,4]. No effective malaria vaccine is currently available, and drug resistance has been implicated in the spread and re-emergence of the disease [5-7]. Artemisinin drugs, an essential component of treatment for multidrug-resistant falciparum malaria, have recently shown signs of decreased efficacy in combination drug therapy [8-12].

With the availability of complete genome sequences of *Plasmodium*, humans and *Anopheles*-hosts of the parasite, development of novel effective anti malarial drugs was envisioned [13]. However, in spite of eclipse of more than one decade no significant advances have been made in the area of genomics based anti-malarial drug development. *Plasmodium *genome is highly AT rich as compared to human genome and this opens up opportunities for development of DNA-targeting drugs that can specifically interact with parasite DNA and introduce deleterious changes leading to loss of vital genome function and parasite death. Some DNA targeting drugs such as Aminoquinolines, azaterphenyl diamidines, adozelesin and bizelesin shows anti *plasmodium *activity, however the problem of cytotoxicity and potential threat of mutagenesis in human DNA is major limiting factor [14-16].

## Presentation of the hypothesis

Advances in the development of zinc finger nuclease (ZFN) with engineered DNA recognition domains allow us to design and develop nuclease of high target sequence specificity [17-19]. *In vivo *application of ZFN with a single mega recognition sequence leads to target gene modification in *Drosophila*, zebra fish, mice, *Arabidopsis*, rice, tobacco, and other genomes [20-25], with a gene targeting frequency upto 80% of ZFN-transfected cells [26]. Cell-penetrating peptides (CPP) provide a novel mechanism for intracellular macromolecular delivery [27,28], CPP are known to cross the cell plasma membrane in a nonspecific manner and deliver conjugated protein, nucleic acid, or any other cargo to the cytoplasm, nucleus, or mitochondria, depending on the additional signal sequences present [27,29-33].

**Figure 1:**
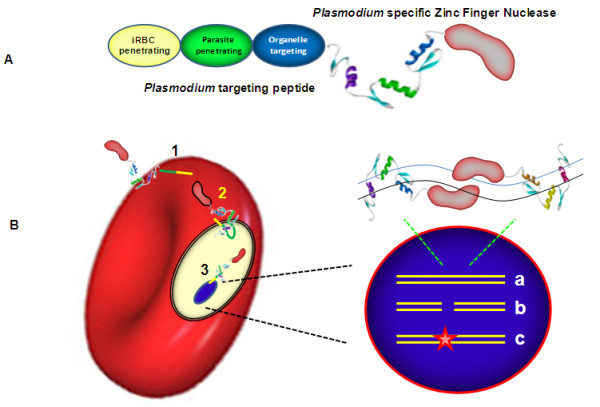
**CPP-ZFN. **A. *Plasmodium*-targeting peptide having signal peptides for infected RBC penetration, merozoite import, and organelle targeting, conjugated with *Plasmodium *specific zinc finger nuclease. B. It is hypothesized that the *Plasmodium*-targeting peptide will cross the membrane barriers of RBC and *Plasmodium*, and ZFN will introduce a double-strand break in the vital gene of the *Plasmodium *genome (a, b), leading to loss of gene function (c) and finally parasite death.

In this article it is believed that a drug from the combination of CPP and ZFN can effectively enter the intracellular parasite, introduce deleterious changes in its genome, and eliminate the parasite from infected cells. Preliminary studies of therapeutic application of ZFN-based drugs against HIV and hepatitis B virus further strengthen the prospects of ZFN-based anti-malarial drug development [26,34]. ZFN mediated disruption of HIV co-receptor CCR5 in human CD4(+) T cells confers resistance to HIV-1 infection [22,26,35]. While ZFN therapeutic against hepatitis B virus intended to directly target and inactivate episomal DNA viral genomes [34]. In the present article, the use of ZFN to specifically target the *Plasmodium *genome with the aim to knock out vital parasite genes is proposed (Figure 1).

### Key hypothesis/assumptions

1. ZFN will specifically target the *Plasmodium *genome.

2. ZFN can cross the plasma membrane barrier of the host and *Plasmodium *cell.

3. DNA double-strand break will kill *Plasmodium*.

## Testing of the hypothesis

### Designing specific ZFN

ZFN can be designed specifically against any gene in any genome [21,22,36-38]. C2H2-type zinc finger proteins consist of an array of protein fingers stabilized by zinc ion, with each finger recognizing a specific triplet of DNA sequence [17]. Protein fingers specific for more than 45 triplets are known and have a modular nature, with freedom to change number of fingers, which makes the development of customized ZFP against diverse target DNA sequence feasible (Additional file 1). A combination of 6 fingers recognizes a DNA sequence of 18 nucleotides that have a probability of occurring after 4^18 ^nucleotides, much more than the human genome size (3000 MB). Hence, a ZFN designed against the *Plasmodium *genome have negligible probability of randomly occurring in the human genome. ZFN designing and validation tools, and CompoZr^® ^custom ZFN designing, assembling, and validation service, reduce the time required for the development of ZFN against new targets [39-42].

### Selection of ZFN target sites

Availability of complete genome sequences have helped identify different anti-malarial drug targets, such as histone deacetylase, aspartic proteases or plasmepsins, aminopeptidases, and the purine salvage enzyme hypoxanthine-xanthine-guanine phosphoribosyltransferase, within the parasite [43-46]. However, limited knowledge on the structure of most of these genes, and on the conservation of basic eukaryotic cellular machinery in *Plasmodium *and humans, hampers the conventional enzyme-targeting drug design procedures [47]. The *Plasmodium *genome is highly AT rich, in contrast to the human genome that is GC-rich; this provides considerable sequence (ZFN target) site diversity even for the structurally and functionally conserved enzymes between *Plasmodium *and humans. ZFN technology can utilize this sequence diversity and overcome the limitations of traditional drug-design approaches, which are confined to parasite-specific metabolic pathways. Moreover, ZFN can be designed against the target site conserved across different *Plasmodium *species (Additional file 2).

### Delivery of ZFN to the target site

Discovery of cell-penetrating peptides provides a novel way of delivering therapeutic molecules to the target cell or organelles [27-29,31,48-51]. A major challenge in ZFN delivery is developing a peptide that can transfer conjugated ZFN through cell membranes of *Plasmodium*-infected RBC (iRBC), the parasite, parasitophorous vacuole, and its organelles (apicoplast, mitochondria, or nucleus).

#### Delivery to RBCs

CPP are generally considered to deliver cargo to cells in a nonspecific manner [51]. iRBCs have different surface proteins and charge that can be utilized for making strategies for specific ZFN delivery. As a matter of evidence, P1 peptides specifically target iRBCs [52], and DPT-sh1/sh2 CPP specifically recognize and deliver conjugated heterologous proteins to iRBCs [53].

#### Translocation from host cell cytoplasm to *Plasmodium*

Following CPP-mediated ZFN delivery to RBCs, the next challenge is to cross the parasite cell membrane and interact with its DNA. CPP-mediated protein delivery to different intracellular organelles such as the nucleus and mitochondria have been reported [54,55]. Intracellular *Plasmodium *imports a number of proteins, such as heme, δ**-**aminolaevulinate (ALA), and peroxiredoxin 2, from the host cytoplasm [56-58], which could be a valuable source of signal sequences for translocation of ZFN from the host cytoplasm to the parasite.

#### Targeting to *Plasmodium *organelles

Once ZFN enters the *Plasmodium*, it has to be directed towards any of its organelles-apicoplast, mitochondria, or nucleus-which have their own genome. Use of the yeast GAL4 nuclear localization signal leads to translocation of green florescent protein (GFP) to *Plasmodium *nucleus and shows conservation of nuclear localization signal in *Plasmodium *and other eukaryotes [59]. GFP was fused with the N-terminal sequence of heat shock protein (PfHsp); PfHsp-GFP was targeted to *Plasmodium *mitochondria [60]; and apicoplast targeting was achieved by using the signal sequence of acyl carrier protein [61-63].

Availability of iRBCs penetrating peptide [53]; *Plasmodium *import proteins [56-58]; and *Plasmodium *apicoplast, mitochondria, and nucleus targeting signal sequences [59-63] provides building blocks for the development of a *Plasmodium*-targeting, multiple-membrane-traversing peptide. Following the successful fusion of CPP with mitochondrial- and nuclear-targeting peptides [54,64,65], fusion of CPP with 1 more *Plasmodium *cell membrane translocation peptide appears to be achievable.

### Hydrolysis of double-stranded DNA

A ZFN-induced double-strand break (DSB) triggers either of the 2 DNA repair processes: error-prone non-homologous end joining (NHEJ) and error-proof homology-dependent repair (HDR). Genetic and biochemical evidence support the hypothesis that NHEJ and HR are 2 independent and competing mechanisms for DSB repair in diploid organisms[66,67]. Being in the haploid stage in humans, HDR must be absent in *Plasmodium *and the error-prone repair mechanism of NHEJ often results in localized mutations due to deletion and/or insertion of short sequences at the DSB site, thereby resulting in disruption of functional gene expression in *Plasmodium*. Moreover, AT-specific DNA alkylating drugs have previously shown anti *P. falciparum *activity and recent advances in ZFN directed multiple DSB resulting in chromosomal deletions further strengthens the prospects of DNA targeting anti malaria drug [68-70].

### Experimental validation of ZFN therapeutic efficacy

An *in silico *designed and *in vitro *validated ZFN is to be evaluated for bioavailability, potency, *in vivo *localization and their specificity for parasite over human DNA. A microfluorimetric method using PicoGreen^® ^can be used for assessing susceptibility of parasites to CPP-ZFN compounds [71,72]. Since all ZFN consist of nuclease domain of FokI restriction enzyme so FokI antisera can be used for immuno-localization and quantification of ZFN [73]. Alternatively ZFN are designed to have an additional small protein tag, such as Flag or His tag and subsequently detected by fusion tag specific antibodies [74]. The quantification of DSB repair loci induced by ZFN provide genotoxicity assay, to test any off target effect of parasite specific ZFN over human genome [75,76].

### Implications of the hypothesis

The major advantage of ZFN technology is that it enormously increases the number of drug targets because it can utilize the vast sequence diversity among structurally and functionally conserved enzymes of human and *Plasmodium *proteins. Prolonged use of any drug forces the evolution of drug-resistant parasite strains, and ZFN would not be an exception. Another advantage of ZFN technology is that it allows convenient development of new ZFN against other target sites (18 bp) in the same gene. The resulting new drug will be effective even against resistant strains, providing ample alternatives for drug resistance management.

Unlike common drugs that directly inhibit the target protein, ZFN technology is focused on inhibiting the synthesis of a functional target protein; thus, its effect will be slower than inhibitor drugs. This constraint may not be a concern as ZFN utilizes the difference between human and *Plasmodium *gene sequences, so every vital gene becomes a potential drug target. Selection of genomic locus coding for short half-life will greatly enhance the response time of ZFN-based drugs.

Availability of newer drug targets to virtually all *Plasmodium *genes provides the opportunity to find conserved sites in vital genes and develop a single drug against all malarial infections, including multidrug-resistant strains, for worldwide use. CPP-mediated protein delivery is an established method that can be used for delivery of the newly designed ZFN. CPP-ZFN promises to be a safe and sustainable drug for malaria intervention.

## Conflict of interests

The authors declare that they have no competing interests.

## Authors' contributions

VN: conceived the idea. AV, SS, and VN: involved in intellectual discussions, formulated the hypothesis, and wrote the manuscript. SS and VN: created graphical presentations and designed ZFN against the target site. All authors read and approved the final manuscript.

## Supplementary Material

Additional file 1 **Designing of target-site-specific zinc finger nuclease (ZFN) through modular assembly**. With the target site specific ZFP designing by incorporating zinc finger helices, linkers, N and C-terminal fixed sequences, ZFP binding to target site can be validated by gel shift assay. As ZFP do not have nuclease activity it does not cut the target sequence. Incorporation of nuclease domain of FokI constitutes the functional ZFN that can be validated by *in vitro *digestion of DNA with target sequence. Click here for file

Additional file 2 **Designing of zinc finger nuclease (ZFN) against a conserved site in lactate dehydrogenase genes of *P. falciparum *and *P. vivax***. Two ZFN designed on opposite strands will introduce a nick in the spacer region, leading to a double-strand break. ELISA data of multi-target specificity assay for all triplets, black bars represent target oligonucleotides, while white bars represent oligonucleotide pools with a particular 5' nucleotide. The height of each bar represents the relative specificity of the protein for each target [42]. Click here for file
