# Supplementary figures and images for: CPP-ZFN: A potential DNA-targeting anti-malarial drug
Source: Malar J. 2010 Sep 16;9:258. doi: 10.1186/1475-2875-9-258 (PMC2949742; doi:10.1186/1475-2875-9-258)

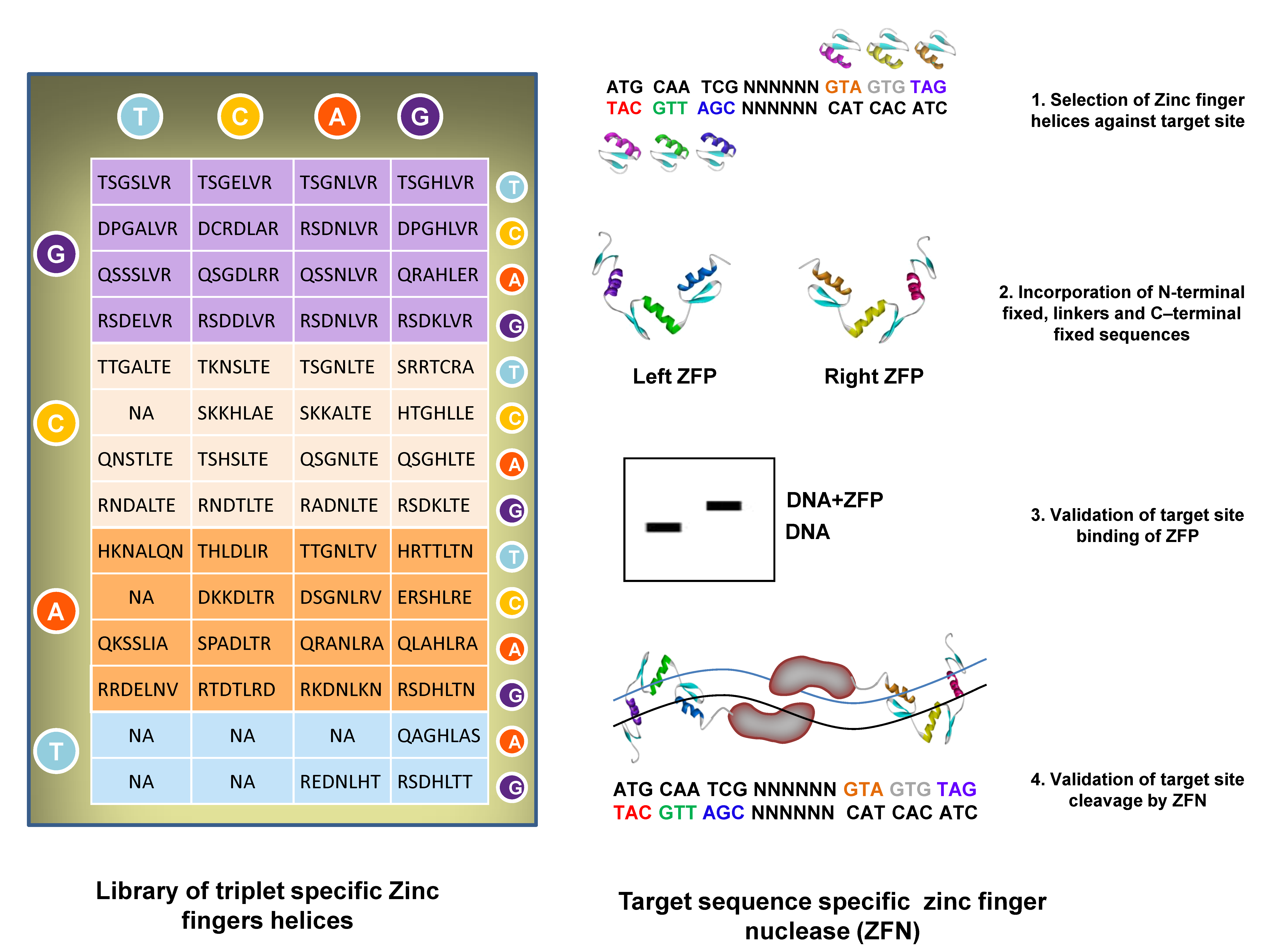

Supplement: Additional file 1 — Designing of target-site-specific zinc finger nuclease (ZFN) through modular assembly. With the target site specific ZFP designing by incorporating zinc finger helices, linkers, N and C-terminal fixed sequences, ZFP binding to target site can be validated by gel shift assay. As ZFP do not have nuclease activity it does not cut the target sequence. Incorporation of nuclease domain of FokI constitutes the functional ZFN that can be validated by in vitro digestion of DNA with target sequence. [file 1475-2875-9-258-S1.TIFF]

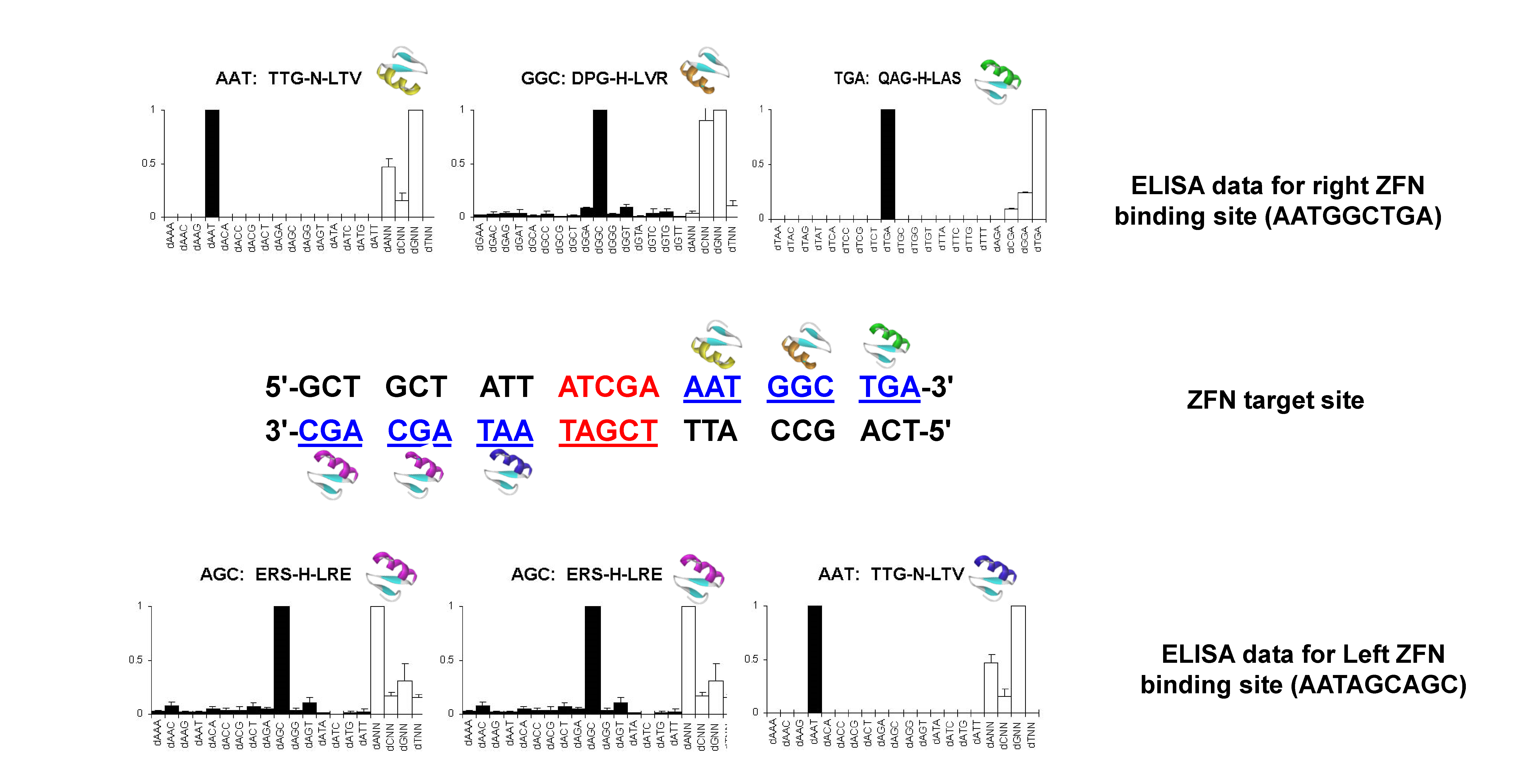

Supplement: Additional file 2 — Designing of zinc finger nuclease (ZFN) against a conserved site in lactate dehydrogenase genes of P. falciparum and P. vivax. Two ZFN designed on opposite strands will introduce a nick in the spacer region, leading to a double-strand break. ELISA data of multi-target specificity assay for all triplets, black bars represent target oligonucleotides, while white bars represent oligonucleotide pools with a particular 5' nucleotide. The height of each bar represents the relative specificity of the protein for each target [42]. [file 1475-2875-9-258-S2.TIFF]
